# Further Success with Potassii Nitras

**Published:** 1890-06

**Authors:** L. W. Cock

**Affiliations:** Laredo, Texas


### Correspondence.

### FURTHER SUCCESS WITH POTASSII NITRAS IN CHILL.

LAREDO, Texas, April 15, 1890.

*Editor Daniel's Texas Medical Journal:*

I wish to add my testimony to that of Dr. Hunter, regarding the value of pot. nit. I was called, on March 22, to see a gentleman, a recent resident of the Mississippi valley. Gave a history of chronic malaria for past four years. Condition was intensely jaundiced. At the time of my first visit he was suffering severely with hepatic colic. A hypodermic of morp. and atropia gave him relief; ordered a saline purgative. Had no further trouble until next alternate day, when I was hastily summoned to find him suffering with a decided chill, hepatic colic with plainly marked fecal obstructions in the region of the umbilicus. Gave hypodermic for relief of pain and succeded in removing the obstruction by large enemas of olive oil, followed by salines. Patient passed between forty and sixty gall-stones in twelve hours. Next day gave large doses of quinia per mouth and rectum, but on the following day, at 10 a. m., patient had a severe chill, followed by a temperature of 106° axilla; reduced the temperature by hypodermics of antipyrine. On the next chill day ordered nothing but pot. nit., grs. xxx, at 6 a. m., grs. xxx at chill time. Result, no chill since; jaundice of one year's standing entirely gone; skin cleared up; appetite good; says that he feels better than he felt has for four years. I attribute this result to two things; to potassi nitras and to the matchless and wonderful climate of Laredo. L. W. COCK, M. D.

